# Extinction and the temporal distribution of macroevolutionary bursts

**DOI:** 10.1111/jeb.13741

**Published:** 2020-11-27

**Authors:** Stephen P. De Lisle, David Punzalan, Njal Rollinson, Locke Rowe

**Affiliations:** ^1^ Evolutionary Ecology Unit Department of Biology Lund University Lund Sweden; ^2^ Department of Biology University of Victoria Victoria BC Canada; ^3^ Department of Ecology and Evolutionary Biology University of Toronto Toronto ON Canada; ^4^ School of the Environment University of Toronto Toronto ON Canada; ^5^ Swedish Collegium for Advanced Study Uppsala Sweden

**Keywords:** macroevolution, microevolution, population dynamics, stabilizing selection, stasis paradox, survivorship bias

## Abstract

Phenotypic evolution through deep time is slower than expected from microevolutionary rates. This is the paradox of stasis. Previous models suggest stasis occurs because populations track adaptive peaks that remain relatively stable on million‐year intervals, raising the equally perplexing question of why these large changes are so rare. Here, we consider the possibility that peaks can move more rapidly than populations can adapt, resulting in extinction. We model peak movement with explicit population dynamics, parameterized with published microevolutionary estimates. Allowing extinction greatly increases the parameter space of peak movements that yield the appearance of stasis observed in real data through deep time. Extreme peak displacements, regardless of their frequency, will rarely result in an equivalent degree of trait evolution because of extinction. Thus, larger peak displacements will rarely be inferred using trait data from extant species or observed in fossil records. Our work highlights population ecology as an important contributor to macroevolutionary dynamics, presenting an alternative perspective on the paradox of stasis, where apparent constraint on phenotypic evolution in deep time reflects our restricted view of the subset of earth's lineages that were fortunate enough to reside on relatively stable peaks.

## Graphical abstract

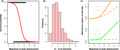

## 1 INTRODUCTION

Phenotypic change can be rapid at microevolutionary timescales (Gotanda et al., 2015; Hendry & Kinnison, 1999; Kinnison & Hendry, 2001), consistent with strong selection (Conner, 2001; Endler, 1987) and abundant genetic variance (Mousseau & Roff, 1987). Yet these microevolutionary processes do not appear to accrue to influence inferred evolutionary rates in deep time (Eldredge & Gould, 1972). This apparent disparity in evolutionary rate across timescales is often referred to as the paradox of macroevolutionary stasis, where ‘stasis’ is relative; observed rates of evolution in deep time are low *relative* to expectations from microevolutionary theory and data. Recent analyses of an impressive data set combining microevolutionary, fossil and comparative data on vertebrate body size have recapitulated the problem of stasis (Estes & Arnold, 2007; Gingerich, 1993, 2001; Uyeda et al., 2011). These new analyses fit models of stabilizing selection towards shifting trait optima and indicate that although evolution can be rapid for a handful of generations, this period of rapid change is followed by a protracted period of relatively slow evolution lasting for over one million years, prior to striking bursts of divergence in deeper time (Arnold, 2014; Uyeda et al., 2011). These new results highlight a fundamental and largely unaddressed question in contemporary evolutionary biology: what processes may account for discrepancies in our inference of evolutionary dynamics between analyses of recent and deep time?

The pattern of limited phenotypic change accruing over long timescales followed by macroevolutionary bursts suggests that some process must constrain the accrual of phenotypic change within populations, as well as among closely related populations. Concomitantly, these results suggest that selection in the wild is net stabilizing, with large changes in the optimum phenotype occurring with exceptional rarity. Despite some indications of considerable temporal variation in the direction of selection (Siepielski et al., 2009, 2011), little is known about the behaviour of adaptive peaks, and previous treatments have typically assumed relatively slow movement (Estes & Arnold, 2007). Identifying why phenotypic change appears constrained over long timescales and hence resolving the ‘paradox of stasis’, remains one of the most important problems to arise from the modern synthesis (Arnold, 2014; Futuyma, 2010; Hansen & Houle, 2004; Pujol et al., 2018; Voje et al., 2018), embodying the fundamental challenge of identifying the microevolutionary processes that can explain patterns of divergence at greater timescales.

Estes and Arnold (2007) analysed a data set (Gingerich, 1993, 2001) of contemporary and historical rates of vertebrate evolution, and found that the only models of phenotypic evolution that seem to explain observed body size evolution across timescales depended primarily on movement of an adaptive peak (or optimum), whereas standing genetic variance (i.e. trait heritability) had little explanatory power (Arnold, 2014). Subsequently, Uyeda et al. (2011) performed a similar analysis after incorporating phylogenetic and additional data. Focusing on several models of peak movement, they concluded that the best‐supported model is one of multiple peak displacements, where displacements of varying magnitude occur relatively infrequently (Uyeda et al., 2011). A recent study by Landis and Schraiber (2017) also supports models of episodic peak movement as the best model to explain patterns of vertebrate body size evolution. A problem that emerges when considering a model with peak movement is that, in real populations, displacements from the optimum phenotype will often be accompanied by reductions in mean absolute fitness and, thus, population size (Haldane, 1937). In the extreme case, frequent or extreme displacements will result in an extinction event. Even under weak selection and modest but persistent peak movement, population size can fall below the replacement rate and the population will eventually go extinct (Bürger & Lynch, 1995; Gomulkiewicz & Holt, 1995; Lynch & Lande, 1993). Although Uyeda et al. (2011) acknowledged that extinction could play a role generating the patterns observed in their data, they did not explicitly consider extinction in their likelihood‐based approach to estimating model fits and parameters or consider how extinction may influence estimates of peak displacements. A previous treatment (Estes & Arnold, 2007) made similar restrictive assumptions regarding the magnitude of between‐generation peak movement, on the grounds that displacements of excessively large magnitudes would result in the extinction of lineages. Yet the importance of extinction for patterns of phenotypic diversity is broadly recognized (Jablonski, 2008), and moreover, the observation that the vast majority of biodiversity is now extinct (van Valen, 1973) suggests extinction has played a critical role in shaping the patterns of diversity we see both today and in the past. This also highlights an important shortcoming of many studies attempting to draw conclusions regarding the behaviour of adaptive peaks, simply from phenotypic (both neontological and paleontological) divergence data; these provide an incomplete view, restricted to those instances in which a lineage was able to persist despite changes in selective regimes.

In the present study, we attempt to reconcile patterns of morphological stasis with observed variation in selection in the wild, by incorporating microevolutionary processes and population dynamics into macroevolutionary models. Our goal was not to reanalyse the previous studies, critique any specific model of evolution or fit models explicitly to empirical data. Rather our modelling approach attempts to highlight the effects of a general feature of biological populations—extinction—which has rarely been considered in models of phenotypic macroevolution (but see Bokma, 2008; FitzJohn, 2010). We focus on a subset of models of peak movement previously identified as the prime candidates for the observed data (Arnold, 2014). We simulated anagenetic evolution using empirically derived estimates of peak displacement, while simultaneously considering the effect of these displacements on the likelihood of extinction by tracking population dynamics explicitly. Although nonrandom extinction is known to influence our ability to interpret macroevolutionary models (e.g. when trait values correlate with extinction; Maddison, 2006), and demography is known to impose constraint on microevolutionary change (Gomulkiewicz & Houle, 2009), the potential effects of extinction on our ability to estimate the dynamics of the adaptive landscape are currently unclear. We show that (a) available estimates of phenotypic selection coefficients from temporally replicated studies in the wild indicate that displacements of the optimum are far more commonplace and severe than inferred from previous analyses of phenotypic change and (b) incorporating extinction explicitly into macroevolutionary models of peak movement indicate that lineage loss could contribute substantially to apparent patterns of morphological stasis. Put simply, extinction can lead to low probability that any large peak displacement can result fully in phenotypic change, even if such shifts are commonplace. Our work suggests that inference on the movement of adaptive peaks using observed phenotypic data alone fail to capture the fact that lineage loss may erase the history of rapid or severe peak displacements. Moreover, our work demonstrates that explicit integration of population ecology may shed light on patterns of phenotypic evolution in deep time.

## 2 METHODS

### 2.1 Basic approach to simulating phenotypic evolution and population size

We simulated the evolution of a quantitative trait in replicate populations, where populations were subject to several different scenarios determined by a range of evolutionary genetic parameters. We focus on anagenetic evolutionary change (our model contains no speciation process). Our simulations were demographically explicit, although for all parameter values we contrast two categories of model: one where population size is permitted to decrease to extinction under maladaptation, and another where population size was ‘rescued’ artificially and kept above a threshold minimum size. This later treatment, although biologically unrealistic, allows us to contrast the effects of extinction across otherwise identically parameterized simulations. We note this later treatment is analogous to typical approaches to modelling phenotypic macroevolution, which are typically agnostic to the demographic effects of maladaptation. We assumed phenotypic selection acting on a single, continuously distributed trait, *z*, with a population mean phenotype, $\bar{z}$, experiencing selection approximated by a Gaussian fitness function (roughly corresponding to the ‘adaptive landscape’; Arnold et al., 2001; Lande, 1979) with a width, ω, with the position of a single optimum located at θ, and described by:

(1) $$W\left(z\right)=\exp\left[\frac{‐{\left(z‐\theta \right)}^{2}}{2\omega^{2}}\right]$$

and we can approximate evolutionary change on this fitness surface via

(2) $$\Delta \bar{z}\approx h^{2}\sigma^{2}\left(\bar{z}‐\theta \right)/\left(\sigma^{2}+\omega^{2}\right)$$

(see (Estes & Arnold, 2007; Lande, 1979)) where *σ* ^2^ equals the phenotypic variance. We simulated evolution of $\bar{z}$ for up to 10,000,000 generations while allowing *θ* to vary in position on a generation‐by‐generation basis (i.e. assuming discrete time with nonoverlapping generations). The behaviour of θ was governed by processes simulating either Brownian motion of the optimum or peak displacement with (potentially) multiple bursts (see Uyeda et al., 2011 and below). In addition to tracking phenotypic evolution, at every generation, *t*, we allowed population size (*N*) to change according to the average fitness of the population, $\bar{W_{t}}$, which depends on the phenotypic distribution relative to the optimum following

(3) $$\bar{W_{t}}=W_{\max}\exp\left[\frac{‐d^{2}}{2\left(\sigma^{2}+\omega^{2}\right)}\right]$$

where *W*_max_ corresponds to maximum absolute fitness, *σ* ^2^ equals the phenotypic variance, and $d^{2}={\left(\bar{z}‐\theta \right)}^{2}$ (Gomulkiewicz & Holt, 1995, Equation 6; Estes & Arnold, 2007, Equation 2) is the distance a population is from its optimum trait value. This yields density‐independent population growth,

(4) $$N_{t+1}=N_{t}\bar{W_{t}}$$

In our simulations of density‐independent population growth, we capped population size at 10 million to prevent geometric expansion to numbers unrealistic for most metazoans.

To model the more realistic scenario of density‐dependent population growth associated with phenotypic evolution, we assumed (logistic) population growth, *r*, is a function of the maximum growth rate ln(*W*_max_) and the distance to the new optimum, described by:

(5) $$r=\ln\left(W_{\max}\right)\times \left(1‐N/K\right)‐\left(d^{2}+\sigma^{2}\right)/2\left(\omega^{2}+\sigma^{2}\right)$$

which can be used to describe demographic change in our model by

(6) $$N_{t+1}=N_{t}\exp\left(r\right)$$

after Lynch and Lande (1993, Equation 2), and incorporating load on population growth introduced by phenotypic variance (*σ* ^2^) (see Kirkpatrick and Barton (1997, Equation 7)).

We assumed that, under a given selection scenario (i.e. for all replicates for a set of parameters, for the duration of the simulated interval) the shape of the fitness function remained constant. In the models considered, the response to selection depended on the available genetic variance (expressed as heritability), *h^2^*.

We focused on two classes of models of peak movement: one that invokes movement of the optimum at relatively constant rate: the Brownian motion of the optimum (Felsenstein, 1988) and another that envisions more sporadic movement: the peak displacement or ‘burst’ model (sensu Estes & Arnold, 2007; Uyeda et al., 2011). To estimate the impact of extinction, we contrasted each model of peak movement when extinction was allowed and when extinction was prevented. In our simulations, extinction was prevented by simply re‐seeding the population with some minimum *N* if the population size fell to zero, or below some critical threshold. Because the multiple‐burst model has been a focus of past work and because similar conclusions of extinction effects are reached under a Brownian motion model, we present the Brownian motion results in the supplemental material.

Although past workers have made use of explicit likelihood functions for alternative models of peak movement that allow comparisons with observed data (Estes & Arnold, 2007; Uyeda et al., 2011), we aim to explore the qualitative effects of peak movement on population dynamics to examine different scenarios that succeed or fail to generate the characteristic pattern of relative stasis, followed by bursts of change (i.e. qualitatively resembling the ‘blunderbuss’ pattern observed by Uyeda et al., 2011). In the models of Brownian motion of the optimum, at each time step (*t* + 1), the position of θ was equal to its position at *t*, plus a deviation with expected mean = 0 and $\text{variance}=\sigma_{\theta }^{2}$. Brownian motion models with (BME) and without extinction (BM) differed only in that the latter allowed the populations to be rescued whenever the population fell below *N* = 50.

To model a scenario where the optimum experiences displacement less frequently than at every generation, the value of *θ* at a given time step was determined by its previous position but potentially also by a displacement that occurred with some probability. As in Uyeda et al. (2011), the probability of a displacement event occurring was modelled as a Poisson process, determined by the parameter, *λ*, or the average expected number of displacement events per generation. In instances where a displacement event occurred, its magnitude was drawn from a normal distribution with mean = 0 and $\text{variance}=\sigma_{\theta }^{2}$. As above, displaced optimum models with (DOE) and without extinction (DO) were distinguished by allowing the latter populations to be rescued whenever *N* < 50. Note that both the probability of a peak displacement λ and variance of peak displacements $\sigma_{\theta }^{2}$ together determine the total rate of peak movement in this model. The distribution of peak movements after a given amount of time is *N*(0, $m\sigma_{\theta }^{2}$), where *m* is the expected number of peak displacements occurring during a time period (Uyeda et al., 2011). As such, peak displacements that are large but extremely rare result in a slow average rate of peak movement through time, whereas frequent displacements of moderate magnitude would result in a higher average rate of peak movement.

### 2.2 Model parameters

Whenever possible and appropriate, we used parameter values that are similar or the same as in past work (Estes & Arnold, 2007; Uyeda et al., 2011). One major difference, however, is that we used a data set of phenotypic selection gradients (Lande & Arnold, 1983) in temporally replicated studies compiled by Siepielski et al. (2011) to calculate the empirical distribution of displacement distances. For all studies in the database that recorded both standardized linear, *β*, and negative nonlinear (corresponding to approximately stabilizing) selection gradients, *γ*, we calculated the distance to the optimum within each selective episode as:

(7) $$d=\left|\bar{z}‐\theta \right|\approx \left|\beta /(‐\gamma )\right|$$

(Phillips & Arnold, 1989, Eqn 11; Estes & Arnold, 2007, Equation 7). By convention, gradients were normalized to a sample‐specific mean of 0 and standard deviation of 1 (Lande & Arnold, 1983). The standardized selection gradients allow estimation of the relative distance between $\bar{z}$ and *θ* within a given *i*th temporal replicate or ‘episode’, but provide no direct information on the actual values/coordinates of *θ*. Nonetheless, under the assumption that at the *i*th episode, $\bar{z}$ is closer to its optimum than it was at episode *i *− 1, the difference in absolute distances, Δ*d*, between a given pair of successive replicates provides a proxy for the total displacement of the optimum between episodes. The variance of the distribution of Δ*d*, $\sigma_{\theta }^{2}$, was used to parameterize peak movement in the simulations, assuming a normal distribution of possible displacements as in the burst models of Uyeda et al. (2011). We relaxed this assumption of normality by also running the same models but with peak displacements drawn from the empirical estimates of Δ*d*. Implicit in our use of the empirical data to approximate displacements of θ is that variation in selection reflects movement of the optimum, and not temporal variability in the phenotypic distribution. We recognize that the Siepielski et al. (2009) data set may reflect both sources of variation and results in unbiased, albeit imperfect, estimates of peak displacements. As a caveat, our approximation of Δ*d* assumes a quadratic fitness surface and does not explicitly incorporate measurement error (Morrissey & Hadfield, 2012), which is unreported for many studies. Following these caveats and Estes and Arnold's (2007) treatment of similar data, we excluded several observations corresponding to questionably large values of *θ* (i.e.> |35| standard deviations) that most likely reflect gradients associated with large estimation errors.

Each simulation began (i.e. at *t* = 0) with a population mean phenotype, $\bar{z}$ = 0, and phenotypic variance, $\sigma_{p}^{2}=1$. We arbitrarily set *W*_max_ = 1.5, which corresponds to rapid exponential population growth (Malthusian *r* = .41) for populations residing at the optimum. Variation in *W*_max_ had little effect on conclusions under density‐dependent population growth, as this growth rate is rarely experienced (e.g. a small population residing at its optimum phenotype), and also had little effect under density‐independent growth (Figure S1). For each simulated population, carrying capacity (*K*) was randomly drawn from a range of 10,561 < *K* < 12,259, based on estimates for vertebrates (see table 2 in Reed et al., 2003), and each simulation began with a population at carrying capacity (i.e. *N* = *K*). Preliminary runs when carrying capacity was allowed to vary between values an order of magnitude lower or higher (1,000 < *K* < 120,000) generated plots virtually indistinguishable from those obtained using the empirically informed range, suggesting that variance in lineage‐specific carrying capacity has little bearing on patterns of divergence.

For each population, heritability (*h^2^*) was drawn from another empirical data set on vertebrate body size (Hansen et al., 2011), and we assumed a constant *h^2^* throughout a given simulation replicate. For all simulations, we assumed an adaptive landscape with a width of *ω* ^2^ = 3, comparable to median values from previous synthetic studies (Estes & Arnold, 2007). Although we did not exhaustively explore the entire parameter space, the qualitative results of the simulations (i.e. the shape of the plots; see Appendix S1) were generally robust to changes in parameters, though notable exceptions and their interpretation are addressed in the Results and Discussion. We note our scale of divergence (in units of phenotypic standard deviation) differs from the one used by Uyeda et al. (2011), who reported divergence in units of log‐differences in body size. We chose a variance‐standardized scale in order to relate our models to contemporary estimates of selection, which are reported in units of phenotypic standard deviations.

Obtaining an empirical benchmark for the frequency and magnitude of shifts in the optimum is difficult. Indeed, the very premise of this paper is that estimates of λ and $\sigma_{\theta }^{2}$ from some empirical data sets suggest peak displacements are extremely rare, seemingly at odds with estimates of selection in the wild. Because the expected distribution of phenotypic divergence under a multiple‐burst model is *N*(0, $m\sigma_{\theta }^{2}$), where the number of bursts *m* in a given timescale *t* is given by $m=\sum_{t}\lambda$, λ and *σ_θ_* jointly govern evolutionary rate in the model. We bracketed values of the rate parameters in our simulations based on two empirically informed upper and lower bounds. For the lower bound, we assumed λ = 10^–7^ and *σ_θ_* = 3, approximating the values estimated in other studies (Arnold, 2014; Uyeda et al., 2011), which corresponds to an average waiting time of 10 million years for a modest shift in the optimum trait value. Note that because we are working in time units of generations, as opposed to years as in these past studies, our lower bound is conservative; most vertebrates have generation times far in excess of 1 year. Although no empirical benchmark for an upper bound for *λ* exists in the literature, an approximation can be obtained from our empirical sample of Δ*d*, where values of Δ*d* > 3 sds across a generation are observed in 48 out of 227 cases, indicating *λ* on the order of 10^–1^. Because this upper bound is likely biased upwards due to sampling error and regardless is approaching a Brownian Motion model (see Appendix S1) we capped our upper bound of *λ* at 10^–4^. Finally, we note that although rate parameters determine the magnitude of the effect of extinction on patterns of divergence across lineages, for any specific lineage the magnitude of a peak shift (when it occurs) alone determines the severity of the bias arising from extinction (see Results).

We compared the distributions (variances) of phenotypic divergence at the end of each simulation run using Levene's test for equality of variances, and we also plot the accrual of phenotypic variance through time. For each model, we simulated 500 replicate lineages (100 for simulations run to 10^7^ generations). Simulations were implemented using the R base package (R Core Team, 2017), and examples of code for our 4 main classes of models (BM, BME, DO, DOE) are available in the Appendix S1. The subsets of data analysed to generate estimates of Δ*d* and from which *h^2^* was sampled are available in the Appendix S1.

## 3 RESULTS

**FIGURE 1:**
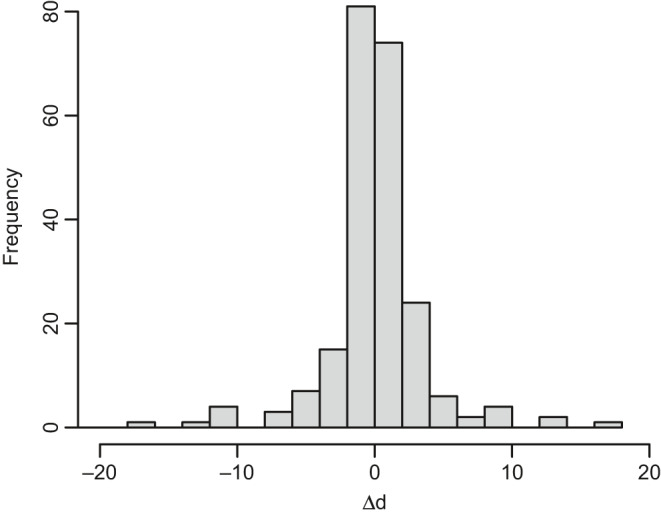
Histogram of estimated temporal (per‐year) changes, Δ*d*, in the displacement of the population mean phenotype from the phenotypic optimum $d=\left|z‐\theta \right|=\left|\beta /(‐\gamma )\right|$. Thus Δ*d* reflects, in part, among year changes in the optimum phenotype observed in extant wild populations. Based on the data set of selection gradients from temporally replicated studies in Siepielski et al. (2009), restricting data to observed instances of negative nonlinear (stabilizing) selection. To aid in visualization, estimates of $\left|\Delta d\right|>20$ are excluded

Our analysis of the empirical data set of temporally replicated selection gradients resulted in 369 estimates of *θ* from 138 studies. From these, we obtained 231 estimates (from 87 studies) of temporal displacements of the optimum, Δ*d*. The distribution of displacements (around zero) of the optimum was roughly symmetric (Figure 1) around zero, with a Laplace distribution with a standard deviation of 12.98. Although the median magnitude (|Δ*d*|) of 1.21*σ* (mean = 3.92*σ*, standard error = 0.82) is comparable to the estimates for *θ* in Estes and Arnold (2007), our empirical estimate of the standard deviation of Δ*d* is far higher than estimates of the variation in Δ*d* inferred in past empirical studies. Specifically, ML estimates of the magnitude of peak displacement from ref 8 correspond to a standard deviation of Δ*d* of approximately 3 phenotypic standard deviations (Arnold, 2014), an order of magnitude less than the empirically observed value.

**FIGURE 2:**
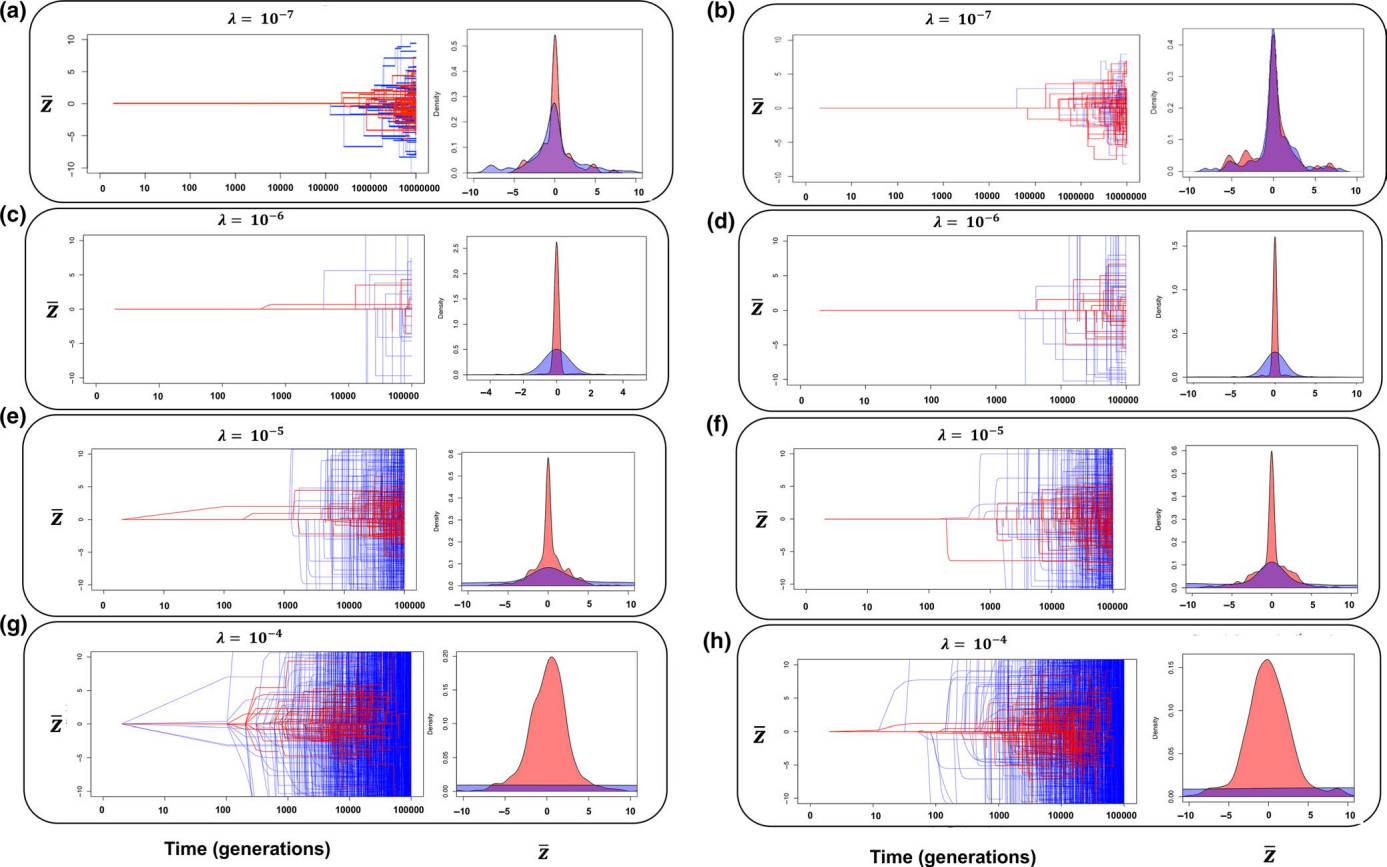
Displaced optimum models (DO, blue; and DOE, red), assuming *σ_θ_* = 3 (Panel a, b) or *σ_θ_* = 13 (the empirically observed value) at different assumed probabilities of a peak displacement, *λ*. Lambda represents the probability that the optimum shifts in a given generation, with 1/*λ* giving the expected wait time for an optimum shift in units of generations. In panels a, c, e and g, population growth was assumed to be density‐dependent, and in panels b, d, f and h, population growth was assumed to be density‐independent. DOE models allow the possibility of extinction for maladapted populations, whereas populations in DO simulations are ‘rescued’ from potential extinction (see text). Right sub‐panels indicate the phenotypic distributions at the end of the simulations; either at extinction or 10^5^ generations (10^7^ for panels a and b). Note scale differences in *x*‐axes of right sub‐panels

**FIGURE 3:**
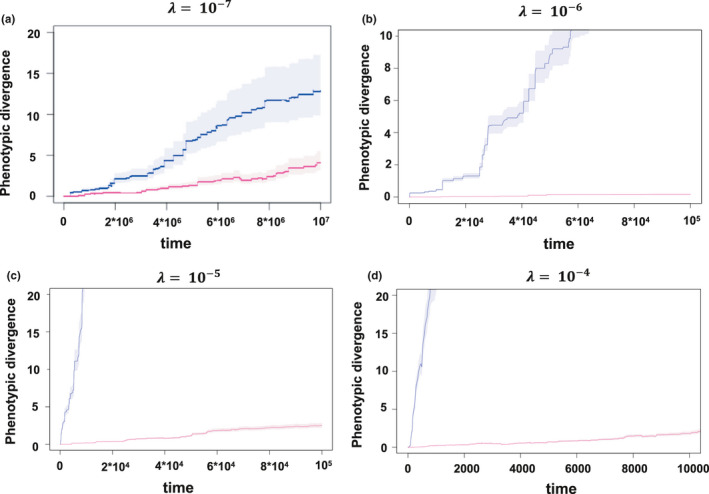
Variance accrual through time in displaced optimum models (DO, blue; and DOE, red), assuming our lower empirically informed benchmark of *σ_θ_* = 3 and *λ* = 10^–7^ (panel a), *σ_θ_* = 13 (panel b, c) at different assumed probabilities of a peak displacement, *λ*. DOE models allow the possibility of extinction for maladapted populations, whereas populations in DO simulations are ‘rescued’ from potential extinction (see text)

Next, we modelled discrete peak displacements that occur with varying frequency, from our lower empirically motivated benchmark of infrequent and modest shifts (i.e. *λ* = 10^–7^, *σ_θ_* = 3; see Uyeda et al. (2011), to three orders of magnitude higher (*λ* = 10^–4^) and with magnitude proportional to the empirically approximated distribution of Δ*d* obtained above. The model yields a narrow band of initial divergence that becomes considerably more distinct when extinction is permitted (Figure 2, left sub‐panels). When peak displacements are assumed to be rare and at our lower empirical benchmark (Figure 2a), extinction has substantial consequences on the amount of divergence observed in deep time (Levene's test: *F*_1,198_ = 11.44, *p* = .0008, Figure 2a right panel). Increasing the value of lambda by one order of magnitude while decreasing the timescale of simulation by two orders of magnitude further illustrates that rare (in this case, relative to the timescale of observation) shifts in the optimum can have striking effects on accumulation of phenotypic change (Levene's test: *F*_1,998_ = 7.85, *p* = .005, Figure 2c). Further increasing the frequency of peak displacements has severe demographic consequences, in turn restricting early divergence (Figure 2e–h), and illustrating the potential for extinction to generate a pattern of apparent evolutionary constraint (all Levene's test: *p* < .0001). When we assume that peak displacements still occur sporadically but with even higher frequency (*λ* = 10^–4^), some key insights are revealed. Extinction becomes critical to shaping the temporal pattern of phenotypic evolution, as divergence becomes otherwise unconstrained over longer timescales (Levene's test: *F*_1,998_ = 737, *p* < 2.2*10^–16^, Figure 2c, right panel). Shorter waiting times between displacements lead predictably to a shortening of the period of relative stasis, and a shorter average longevity of lineages (Figure 2e–g). More frequent displacement events also result in a coarse transition between the period of stasis to the eventual ‘bursts’ of evolution, implying that longer periods of favourable conditions, and therefore more stable population sizes, are more conducive to the formation of patterns to those that have been empirically observed. Noteworthy is that the assumed model of population growth, density dependent versus independent, has little effect on these observed patterns (cf Figure 2a–f). Moreover, although the effects of extinction are most pronounced when peak displacements are frequent and large in magnitude, we observed statistically significant and striking effects of extinction even at our lower benchmark values of *λ* and *σ_θ_* (2, 3, 4 and 2, 3, 4), and these effects persist unless population growth is unbounded towards exceptionally large population sizes (Figure 2b). The substantial effect of including extinction can be readily observed in the accrual of phenotypic variance through time, which is drastically reduced when extinction is permitted (Figure 3a–d), even when peak displacements are exceptionally rare (*λ* = 10^–7^) and the distribution of Δ*d* is reduced to 3 phenotypic standard deviations (Figure 3a). These general effect of extinction on reducing the apparent evolutionary rate holds under a Brownian motion model of peak movement (See Appendix S1).

**FIGURE 4:**
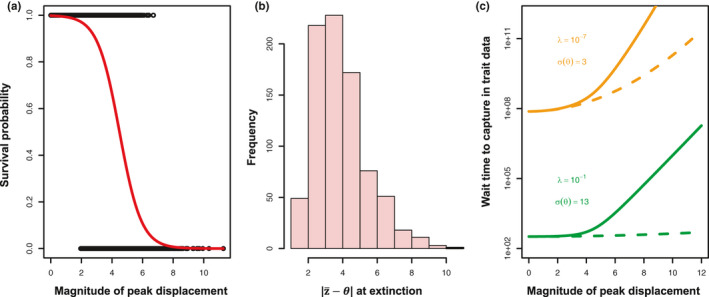
Lineage loss is biased towards large displacements of the optimum, and this can lead to an increase in the wait time to observe peak shifts in trait data. Panel (a) shows the relationship between lineage survival (to 100 generations after the displacement) and the magnitude of the displacement. Panel (b) shows the distribution of distances d each extinct lineage was from its optimum upon extinction, illustrating that large peak displacements are not fully reflected in phenotypic change. Based on simulations where lineages were started at their carrying capacity with a displacement from their optimum sampled from a normal distribution with *σ^2^_θ_* = 9 (changing this distribution has little effect on the observed pattern). Simulations were otherwise parameterized as described in text and assuming density‐dependent growth; the same pattern was found under geometric population growth. Panel (c) shows the probabilities in Panel (a) converted to expected wait times under a displaced optimum model with parameter values set at our upper and lower benchmarks (see text), where wait times are 1/(probability a peak shift × probability of a given magnitude × probability of survival). Dashed lines indicate expectations without extinction. Note log scale of *y*‐axis

These effects of extinction on curtailing the perceived rate of peak movement can be illustrated by considering short‐term survival probability of lineages that experience a single displacement in their optimum phenotype. Displacements beyond approximately 2 within‐population standard deviations are associated with reduced lineage survival over the span of 100 generations (Figure 4a). Lineages that fail to survive have also failed to reach their new optimum (Figure 4b), and so these severe displacements do not result in an equivalent degree of phenotypic change. The probability that a displacement will result in complete phenotypic change approaches zero for displacements beyond approximately 7 standard deviations. Note that these effects are expected regardless of the model of peak movement; a rarity of large peak displacements does not change the likelihood that large displacements lead to extinction when they do occur. Incorporating these survival probabilities into a displaced optimum model of peak movement illustrates extinction's effect on expected wait times to observe a peak shift in trait data (Figure 4c).

Our conclusions from the multiple‐burst model were essentially unchanged when re‐running the model with displacements randomly drawn from the (exponential) empirical distribution of Δ*d* instead of a normal distribution (Figure S3). Although the empirical estimates used to parameterize $\sigma_{\theta }^{2}$ may be biased upwards by sampling error, equivalent conclusions are obtained assuming a value $\sigma_{\theta }^{2}$ that is an order of magnitude lower than the observed value (Levene's test, *F*_1,998_ = 78, *p* < 2.2*10^–16^), and the accrual of variance through time is significantly reduced by extinction at long timescales even when *σ_θ_* is reduced to 3 standard deviations (Figure S2). Only when decreasing $\sigma_{\theta }^{2}$ by over two orders of magnitude from the empirically observed value, to the order of a single phenotypic standard deviation, does the effect of extinction on macroevolutionary divergence disappear (Levene's test, *F*_1,998_ = 0.98, *p* = .32). Yet such a small value of $\sigma_{\theta }^{2}$ is inconsistent with the empirical observation that the divergence is confined to an interval that is several within‐population phenotypic standard deviations wide (Arnold, 2014; Uyeda et al., 2011). Thus, our conclusions regarding the importance of extinction in shaping macroevolutionary patterns appear robust to any potential biases in our empirical estimate of $\sigma_{\theta }^{2}$.

## 4 DISCUSSION

Our analyses illustrate an important and previously underappreciated role for extinction and population dynamics in generating patterns of apparent constraint in macroevolution. We show that in the presence of rapidly moving optima, populations may face extinction while attempting to track their adaptive peak, preventing these rapid peak displacements from being recorded as phenotypic change. Large displacements in trait optima, regardless of their frequency, have exceedingly low probability of resulting in complete phenotypic evolution, thus limiting our ability to infer movements of peaks from trait data alone. As a specific example of extinction's effect on our ability to use trait data to infer movement of corresponding trait optima, we can imagine a clade where the average wait time for a shift in a lineage's optimum of 11 standard deviations is 100 generations. Although this would be expected to be borne out as a very high evolutionary rate, the reduced survival probability associated with such an event (*p* ≈ .0001; Figure 4a) leads to an approximate expected waiting time of one million generations before such an event is captured in trait data. This general effect is expected regardless of whether trait data come from fossil lineages or extant time series. Although the effects of demographic constraint on microevolutionary change are well known (Gomulkiewicz & Houle, 2009; Lynch & Lande, 1993), our work illustrates how these constraints can manifest survivorship bias that leads to apparent patterns of stasis at the macroevolutionary scale. The observation of morphological stasis over macroevolutionary time may not reflect a lack of movement in phenotypic optima, but instead reflect selection bias in phenotypic time series generated by nonrandom extinction, leaving a subset of observable lineages fortunate to have resided on relatively stable optima.

We have focused on two simple models of peak movement, displaced optimum and Brownian motion. These are popular phenomenological models, co‐opted from evolutionary genetics, that avoid the true complexities that must underlie the dynamics of actual adaptive surfaces. A salient conclusion from both models is that the greater the frequency and magnitude of peak shifts, the greater a role extinction will play in shaping patterns of phenotypic evolution across lineages; a conclusion expected to also apply to more complex characterizations of peak movement. Thus, observed patterns of phenotypic divergence in a variety of taxa (Arnold, 2014; Estes & Arnold, 2007; Uyeda et al., 2011) could be partly accounted for by an accrual of changes in many long‐lived lineages fortunate to have experienced only small peak movements, combined with a subset of lineages that, due to abrupt displacements, went extinct before much phenotypic divergence had accumulated.

Using a data set of temporally replicated estimates of phenotypic selection from the wild, we show that the frequency and distribution of peak displacements in extant natural populations are inconsistent with the rarity and modesty of such events as inferred from analysis of observed macroevolutionary phenotypic change that ignores extinction, providing quantitative support to the idea that these past results are indeed paradoxical (as suggested by Uyeda et al., 2011). Concomitantly, such frequent and extreme shifts lead to predicted (in the absence of extinction) macroevolutionary rates that are far more extreme than those observed in empirical data. However, incorporating extinction into these macroevolutionary simulations results in temporal patterns of phenotypic divergence similar to empirical observations, and in all cases resulted in a decrease in the variance in phenotypic change observed in deep time (Figure 2, right panels, Figure 3). Thus, the catastrophic consequences of large peak displacements that were considered “biologically untenable” by Estes and Arnold (2007, p. 237) may, in fact, be the key to reconciling microevolutionary processes with macroevolutionary pattern. Our results suggest that nonrandom extinction plays a crucial role in resolving the stasis paradox; although we frequently observe substantial peak displacements in extant populations (Feya et al., 2015), many of these populations would be expected to face extinction over longer timescales in the face of such maladaptation (e.g. Figure 4), leaving a pattern of apparent morphological stasis over macroevolutionary time despite strong selection observed in extant populations. The relative insensitivity of our results to assumptions of heritability further suggest that extinction can play an important role in patterns of long‐term phenotypic evolution, even when selected traits are evolvable. Moreover, the apparent insensitivity to the form of population growth suggests that our conclusions hold for finite populations.

Extinction will contribute to the appearance of stasis whenever it scales with the frequency or magnitude of peak displacements. Compared with the demographic rescue model in which extinction does not occur, models allowing extinction consistently result in a delay and reduction in phenotypic divergence. This pattern appears to hold under a wide range of values of stabilizing selection (1.5 > *ω* ^2^ > 20), though very strong curvature severely restricts evolution to a narrow range for the entire ‘lifetime’ of the lineage (Figure S5). Similarly, varying the range of heritability did not qualitatively change the importance of extinction. For example, the effect of varying heritability from *h^2^* = 0.9 to *h^2^* = 0.1 was, as expected, primarily to expand or reduce (respectively) the extent of divergence at all timescales (Figure S6). We also explored a “white noise” parameter in a subset of our simulations, in the same manner described by Estes and Arnold (2007). This too had no impact on our conclusions, and omitting this parameter is conservative as it exacerbates the effects of extinction (Figure S7). Thus, a role for extinction in reducing apparent macroevolutionary rate does not appear to be qualitatively dependent on values of heritability, the curvature of the fitness surface, white noise, or even the macroevolutionary/phenomenological model of peak movement. The relatively small role of genetic parameters for explaining broad patterns is consistent with the findings of Estes and Arnold (2007).

Our results are consistent with the observation that extinction is an important contributor to the distribution of Earth's diversity, as most of life that has existed has gone extinct, and suggests that considering extinction events may be critical to understanding shifts in phenotypic optima through the history of life. The extent to which ignoring extinction will impact estimates of peak movements is illustrated in the effects of extinction on the observed variance in phenotypic change, and the accrual of phenotypic change (Figures 2 and 4, left panels, Figure 5). Because both Brownian Motion and Displaced Optimum models rely on the observed distribution of phenotypic divergence in time to obtain estimates of peak movement, where the distribution is treated as *N*(0, $t\sigma_{\theta }^{2}$) (BM) or *N*(0, $m\sigma_{\theta }^{2}$) (DO), any effect of extinction on the realized distribution of phenotypic change, if left unaccounted for, will be borne out in parameter estimates for peak movement that are lower than reality.

Nonetheless, our results suggest the importance of extinction on the inference of peak displacements in data comprising large amalgamations of lineages will depend upon the value of $\sigma_{\theta }^{2}$ and the frequency with which peak displacements occur, because these parameters determine the frequency of large peak displacements that will fail to be fully represented as phenotypic evolution. Yet even at our lower benchmark value of *λ* = 10^–7^ and *σ_θ_* = 3, informed by past work (Arnold, 2014; Uyeda et al., 2011), we find that extinction can play a striking role in limiting the ability of even this slow rate of peak movement to be fully reflected in phenotypic evolution. Thus, our results suggest that estimating rates of peak movement from trait data alone may be problematic at best, even if the true rate of peak movement is very slow relative to our expectations from contemporary microevolutionary studies.

A major challenge in reconciling micro‐ and macroevolution lies in the disparity between processes occurring in a single population versus the dynamics of the suite of interconnected populations constituting a species. Our simulations take the simplistic approach of characterizing evolutionary change assuming a single population per lineage, which has the benefit of allowing existing estimates of microevolutionary parameters (which are measured at the population level) to be incorporated (see also Arnold, 2014; Arnold et al., 2001; Estes & Arnold, 2007; Uyeda et al., 2011). Yet, metapopulation structure across a species range likely has consequences for macroevolutionary dynamics. For example, gene flow among populations within a species can potentially act as a constraint on species‐wide macroevolutionary change (Eldredge et al., 2005; Futuyma, 1987). Further, how selection within populations manifests total selection across a species is unclear (Liebermann & Dudgeon, 1996). Regardless, extinction due to the demographic effects of maladaptation would be expected to result in an apparent reduction of evolutionary rates.

Much of the phenotypic diversity that has ever emerged was probably quickly lost, and extinction has long been recognized as a potentially important force in phenotypic macroevolution; for example, explaining apparent disparities in speciation rates estimated for different timescales in (the ‘Ephemeral Speciation Model’; Auilée et al., 2018; Futuyma, 1987; Rosenblum et al., 2012). Our study complements this view, but with an important distinction: our models do not invoke speciation and only permit anagenetic change. Although the Ephemeral Speciation model (and other models of speciation‐dependent trait evolution; Duchen et al., 2020) emphasizes divergence that was aborted, our models emphasize the importance of extinction for the divergence that cannot accrue in the first place, representing simply one example of the general challenge that extinction imposes for comparative biology. As our ability to infer microevolutionary fitness surface is limited to the distribution of phenotypes we observe, our understanding of the shape and dynamics of macroevolutionary adaptive landscapes is limited by a reliance upon those lineages that have not gone extinct. Thus, extinction poses a challenge for all comparative methods that exploit phenotypic variation to infer nuances of the adaptive landscape, or even estimate rates of phenotypic evolution. Although this issue of extinction has been made especially clear for evolutionary rates of discrete traits (Maddison, 2006), it seems especially germane when the very goal of the evolutionary model is to make a statement on (mal)adaptation (Bartoszek et al., 2012; Hansen, 1997) or the dynamics of macroevolutionary adaptive peaks (Uyeda & Harmon, 2014). The only potential resolution to the challenge extinction poses to comparative biology is to incorporate, whenever possible (e.g. Maddison et al., 2007), the dynamics of lineage accumulation explicitly into macroevolutionary models of trait evolution. Our simulation models represent a first step in this effort to estimate peak movement. Future work further integrating selection, population and macroevolutionary dynamics may present an important way forwards in resolving the paradox of macroevolutionary stasis.

## AUTHOR CONTRIBUTIONS

S.P.D., D.P. and N.R. conceived of the study; all authors contributed to the design of the study; S.P.D., D.P. and N.R. performed the simulations and collected the data; all authors contributed to writing and revising the manuscript.

### Peer Review

The peer review history for this article is available at https://publons.com/publon/10.1111/jeb.13741.

## Supporting information

Appendix S1

## Data Availability

All data and code are presented in the Appendix S1 and are also available on github (https://github.com/spdelisle/Stasis).
