# Supplementary material for: Extinction and the temporal distribution of macroevolutionary bursts
Source: J Evol Biol. 2020 Nov 27;34(2):380–90. doi: 10.1111/jeb.13741 (PMC7983991; doi:10.1111/jeb.13741)
Supplement: Supplementary file 1 — Appendix S1 [file JEB-34-380-s001.zip › jeb13741-sup-0001-Supinfo.docx]

**SUPPLEMENTARY MATERIAL FOR**

**Extinction and the temporal distribution of macroevolutionary bursts**

| **Table S1 parameter space explored in simulations.** Empirical = sampled from distribution of estimates (see text) | | |
| --- | --- | --- |
| parameter | biological meaning | values |
| λ | probability of peak shift | 10^-7^, 10^-6^, 10^-5^, 10^-4^, 1 (Brownian Motion) |
| ω^2^ | width of adaptive landscape | 1.5, 3, 20 |
| σ_θ_^2^ | magnitude of peak shift | .1, .5, 3, 13, empirical |
| *h^2^* | heritability | 0.1, 0.4, 0.9, empirical |
| *K* | carrying capacity | empirical, 10 million (density-independent cap) |
| *W_max_* | absolute fitness at the optimum | 1.2, 1.5, 2, 2.5 |
|  |  |  |

**Figure S1.** Effects of changing W_max_. Top row shows lineage loss under density-dependent population growth with W_max_ = 1.2 (A), 2 (B), and 2.5 (C). Bottom row shows lineage loss under density-independent population growth with W_max_ = 1.2 (D), 2 (E), and 2.5 (F). Simulations were performed as in figure 4, with the exception that simulations assuming density independent population growth were started at N = 100,000.

**S2.** Phenotypic evolution in Displaced Optimum models with (DO, right panel) and without extinction (DOE, left panel) assuming λ = 10^-5^, with σ_θ_^2^ determined by drawing from the empirical distribution of Δ*d.* All other parameters were the same as those in Figure 3A.

**Brownian Motion of the Optimum**

Our BM and BME simulations underline two ways in which Brownian motion models fail to capture empirical patterns at both the micro and macro scale. At a low value of σ_θ_ = 0.1, the pattern of replicated divergence is confined to a relatively narrow band that persists long enough to appear as a period of initial stasis. However, an increase of σ_θ_ to 0.5 results in phenotypic divergence that is much more rapid, and with inconsistent periods of protracted stasis. These patterns appear insensitive to whether or not extinction is permitted when examining plots of divergence through time (Fig. S3A, B, left panels). However, allowing extinction decreases the extent of phenotypic divergence in deep time, and this effect increases with increasing σ_θ_^2^ (Levene’s test for σ_θ_ = 0.5: *F*_1,998_ = 447, *P* < 2.2*10^-16^, Fig. S3B right panel; Levene’s test for σ_θ_ = 0.1: F_1,998_ = 2.04, *P* = 0.15, Fig. S3A right panel; See also Figure S4). Arming the BM models with our empirical estimate of the variance in Δ*d* generates two opposing and equally unrealistic (based on empirical observations) patterns of divergence. When extinction is not allowed, the frequent and persistent movements of the peak result in rapid divergence that almost immediately spans the entire phenotype space; when extinction is permitted, such peak movement results in only moderate divergence before all lineages rapidly go extinct (Figure S3C). Thus, our results not only suggest BM models fail in that they result in too much divergence under realistic parameter values, as noted in the past (Uyeda et al 2011, Arnold 2014), but also that these models fail even with the inclusion of extinction because peak movement can be rapid enough that nearly all lineages fail to survive.


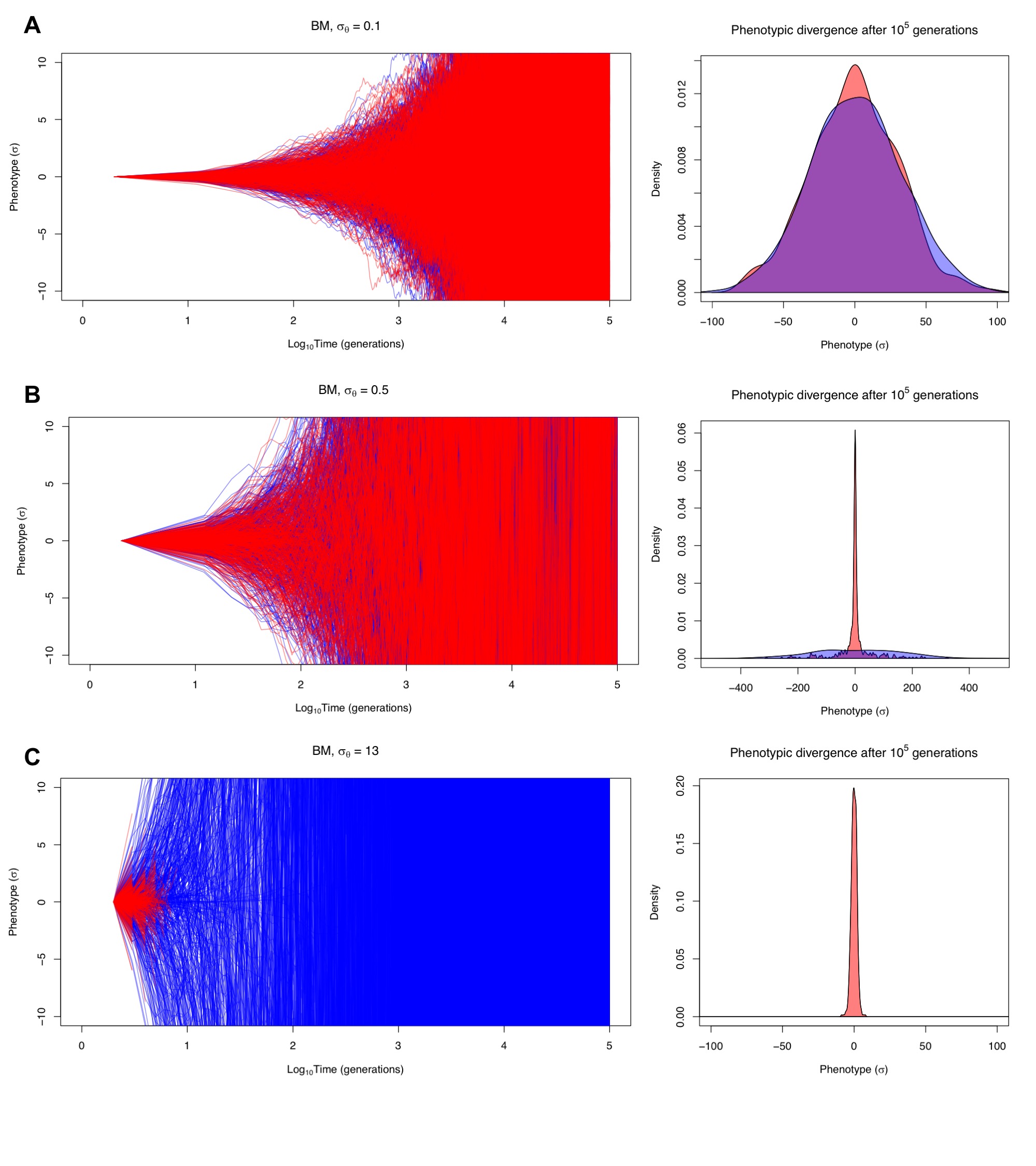


**Figure S3.** Brownian motion models (BM, blue; BME, red) when altering the rate of peak movement, σ_θ_. BME models allow the possibility of extinction for maladapted populations, while populations in BM simulations are ‘rescued’ from potential extinction (see text). A and B represent two low-moderate values of σ_θ_^2^, while C assumes σ_θ_^2^ derived from empirical estimates from wild populations. Right panels indicate the phenotypic distributions at the end of the simulations; either at extinction or 10^5^ generations. Note scale differences in x axes of right panels.


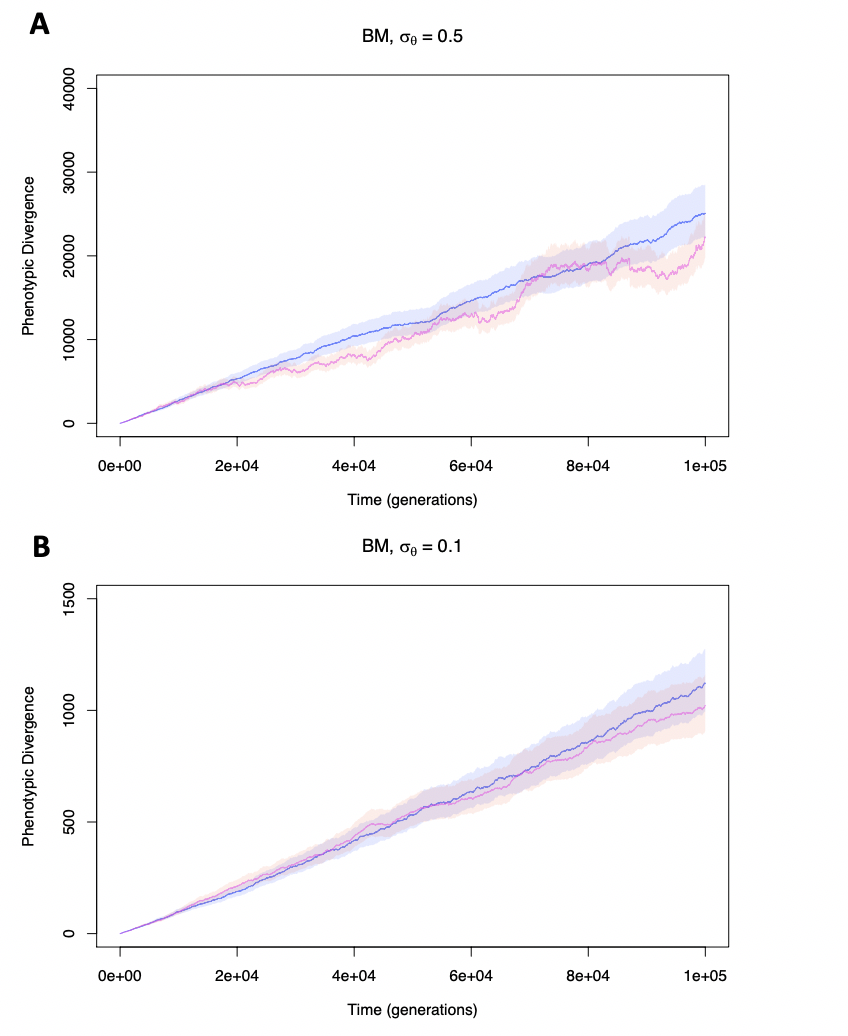


**Figure S4.** Variance accrual through time in Brownian motion models (BM, blue; BME, red) when altering the rate of peak movement, σ_θ_^2^. BME models allow the possibility of extinction for maladapted populations, while populations in BM simulations are ‘rescued’ from potential extinction (see text). A and B represent two low-moderate values of σ_θ_^2^.

**Figure S5.** Effects of changing curvature of the fitness surface on survival probability to 100 generations following a peak shift. Top row shows lineage loss under density-dependent population growth and strength of stabilizing selection = 1.5 (narrow adaptive landscape). Bottom row shows lineage loss under density-dependent population growth and strength of stabilizing selection = 20 (wide adaptive landscape). Simulations were otherwise performed as in figure 4 and described in text.

**Figure S6.** Effects of changing heritability on survival probability to 100 generations following a peak shift. Top row shows lineage loss under density-dependent population growth and heritability of 0.1. Bottom row shows lineage loss assuming heritability of 0.9. Sparse right panel under heritability of 0.9 is because most lineages survived all 100 generations. Simulations were otherwise performed as in figure 4 and described in text.

**Figure S7.** Effects of changing white noise on survival probability to 100 generations following a peak shift. Top row shows lineage loss under density-dependent population growth and white noise = 0. Bottom row shows lineage loss under density-dependent population growth and white noise = 0.5. White noise parameter is a random change in the population mean phenotype. Simulations were otherwise performed as in figure 4 and described in text.
